# The EphB6 Receptor: Kinase-Dead but Very Much Alive

**DOI:** 10.3390/ijms22158211

**Published:** 2021-07-30

**Authors:** Timothy G. Strozen, Jessica C. Sharpe, Evelyn D. Harris, Maruti Uppalapati, Behzad M. Toosi

**Affiliations:** 1 Department of Small Animal Clinical Sciences, Western College of Veterinary Medicine, University of Saskatchewan, Saskatoon, SK S7N 5B4, Canada; timothy.strozen@usask.ca (T.G.S.); jessica.sharpe@usask.ca (J.C.S.); evelyn.harris@usask.ca (E.D.H.); 2 Department of Pathology and Lab Medicine, College of Medicine, University of Saskatchewan, Saskatoon, SK S7N 5E5, Canada; maruti.uppalapati@usask.ca

**Keywords:** Eph receptors, pseudokinase, kinase-independent functions, scaffold, SH2 and SH3 domain binding

## Abstract

The Eph receptor tyrosine kinase member EphB6 is a pseudokinase, and similar to other pseudoenzymes has not attracted an equivalent amount of interest as its enzymatically-active counterparts. However, a greater appreciation for the role pseudoenzymes perform in expanding the repertoire of signals generated by signal transduction systems has fostered more interest in the field. EphB6 acts as a molecular switch that is capable of modulating the signal transduction output of Eph receptor clusters. Although the biological effects of EphB6 activity are well defined, the molecular mechanisms of EphB6 function remain enigmatic. In this review, we use a comparative approach to postulate how EphB6 acts as a scaffold to recruit adaptor proteins to an Eph receptor cluster and how this function is regulated. We suggest that the evolutionary repurposing of EphB6 into a kinase-independent molecular switch in mammals has involved repurposing the kinase activation loop into an SH3 domain-binding site. In addition, we suggest that EphB6 employs the same SAM domain linker and juxtamembrane domain allosteric regulatory mechanisms that are used in kinase-positive Eph receptors to regulate its scaffold function. As a result, although kinase-dead, EphB6 remains a strategically active component of Eph receptor signaling.

## 1. Introduction

**Figure 1:**
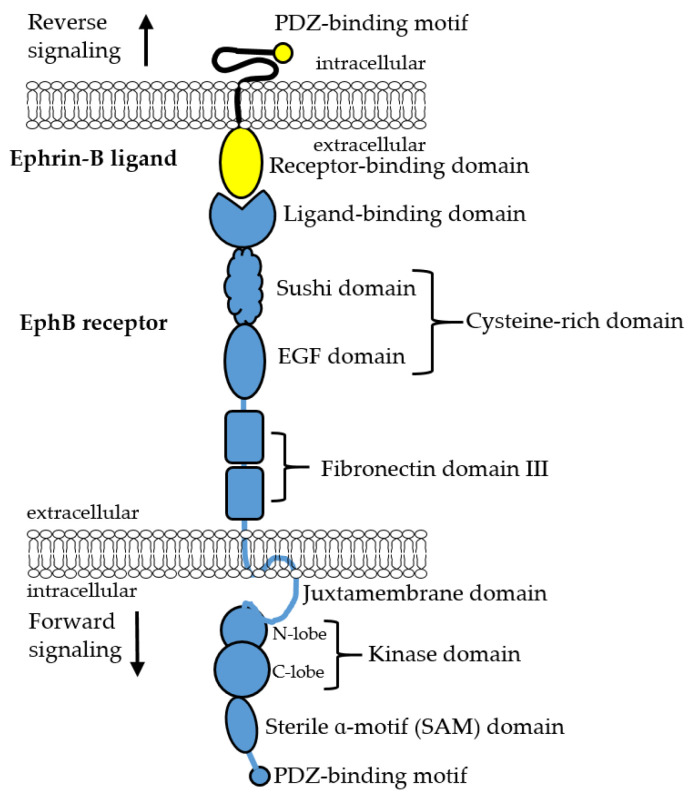
Domain organization of EphB receptors and ephrin-B ligands.

Erythropoietin-producing hepatoma receptor tyrosine kinases (Eph RTKs) and their Eph-receptor interacting (ephrin) ligands serve as a communication conduit between cells, a function that is integral for the evolution of multicellular organisms (reviewed by [1,2]). To date, 16 members of the Eph subfamily of RTKs were identified and classified into two groups, EphA and EphB, of which 14 are present in humans. The EphA (EphA1-8 and EphA10 in humans) receptors predominantly recognize five ephrin A-type ligands that are tethered to the membrane by a glycosylphosphatidylinositol (GPI) linker. The EphB (EphB1-4 and EphB6 in humans) receptors mostly recognize three ephrin B-type ligands that attach to the cell via a transmembrane region and include an intracellular domain with a C-terminal PDZ-binding motif (Figure 1). Together the Eph receptors and their ephrin ligands transduce signals between cells, signals that are paramount to control multiple aspects of cellular and tissue physiology, including cell motility, proliferation and differentiation that are necessary functions both in embryonic development and tissue homeostasis (reviewed by [3]). Not surprisingly, dysregulation of the Eph-ephrin system is exploited to support malignant cell growth, making the Eph-ephrin system a sought-after target for cancer therapy (reviewed by [4,5,6,7]).

All Eph RTKs have a common set of domains (Figure 1). The extracellular portion of Eph RTKs consists of an N-terminal Ligand Binding domain (LBD), a cysteine-rich domain, and two Fibronectin III domains. Following a short transmembrane domain, the intracellular portion of Eph receptors consists of a Juxtamembrane region (JM), a Kinase domain (KD) (if active), a sterile alpha motif (SAM) domain and a PDZ-binding motif (PBM) at its C-terminus. The N-terminal Ligand Binding domain of the Eph receptor is responsible for mediating the interaction with an ephrin via its Receptor Binding domain (RBD). In this manner, activation of Eph RTKs occurs when an Eph receptor on one cell interacts with an ephrin located on an adjacent cell. Cell signaling events that take place upon activation of an Eph receptor are termed “forward signaling”, whereas cell signaling events due to receptor-engaged ephrin ligands are designated as “reverse signaling”.

Upon binding to their ephrin ligands, Eph receptors will form a heterotetramer composed of two receptors and two ephrin ligands, constituting the basic unit of an activated Eph receptor [8]. This interaction tends to be class-dependent with EphA receptors capable of binding ephrin-A ligands and EphB receptors with ephrin-B ligands; however, cross-class promiscuity was verified for some members [9,10], including the ability of EphA4 to recognize ephrin-B2, ephrin-B3 [11] and EphB2 activation by ephrin-A5 [12]. The formation of the Eph receptor/ephrin ligand heterotetramer involves two binding interfaces on the ligand-binding domain of the Eph receptor. The high-affinity ligand-binding pocket uses predominantly non-polar interactions to generate a strong interaction with an ephrin ligand, whereas the low-affinity region uses polar interactions to bind to a second ephrin; likewise, the second Eph receptor forms a weak interaction with the first ephrin ligand and strong interaction with the second [8,13,14]. Curiously, an ephrin-mediated Eph receptor heterotetramer can act to nucleate additional Eph receptors in an ephrin-independent manner to form large oligomeric structures capable of transmitting downstream signals [15,16,17]. In this manner, ephrins located on one cell serve to induce nucleation of Eph receptors in the adjacent cell.

The formation of higher-ordered multimers promotes activation of the kinase-dependent and kinase-independent functions of Eph receptors in the cluster. The kinase-dependent functions involve phosphorylation of tyrosine residues in downstream effector proteins. Kinase-independent functions include the ability to act as a scaffold, whereby phosphorylated Tyr, Ser and Thr residues serve as recognition sites for adaptor proteins containing Src homology-2 (SH2) or phosphotyrosine binding (PTB) domains. Phosphorylation of these highly conserved sites can be accomplished by the tyrosine kinase activity of an adjacent activated Eph receptor or by the activity of other kinases recruited to the cluster. Together, these functions modulate the identity and intensity of forward signals initiated by an Eph receptor cluster.

Pseudoenzymes are protein family homologs that have not retained the ability to catalyze a reaction due to the alteration of specific residues involved in cofactor and/or substrate binding. The incidence of these proteins is widespread, having been identified in approximately 20 protein families that include kinases, phosphatases and proteases (reviewed by [18,19]). High conservation of amino acid sequence and/or structure of pseudoenzymes, when compared to their enzymatically-active counterparts, suggests that these catalytically-inactive family members perform integral roles in cell biology across all kingdoms of life. Current theories propose that pseudoenzymes evolved from genetic duplication of conventional enzymes where the ancestral protein had both catalytic and non-catalytic functions, and the pseudoenzyme retained its non-catalytic mechanisms [20,21]. The human Eph receptors are no exception by including two pseudokinases EphA10 and EphB6 [22,23,24]. These proteins have the same Eph RTK domain organization as other family members yet harbor specific changes in the kinase domain that are required for tyrosine kinase activity. In this review, we focused solely on the intracellular domain of the EphB6 pseudokinase. Although a significant amount of literature has described the biological function of EphB6 in both normal and disease states, there is limited mechanistic information regarding how it performs these cellular functions. As a result, we have used a comparative approach to delve into the potential mechanistic aspects of EphB6, focusing solely on the intracellular portion of the protein that transduces downstream signals.

EphB6 is a protein that is expressed in all tissues and was shown to be important to maintain physiological homeostasis in areas such as the kidney [25], vascular smooth muscle [26] and immune system T-cells [27,28,29,30,31] with the highest expression levels of EphB6 observed in the brain, pancreas and thymus [24,32]. The ephrins that serve as ligands for EphB6 are ephrin-B1 [33] and ephrin-B2 [34]. EphB6 can be phosphorylated via interactions with Fyn [35], EphB1 [33] or EphB4 [36].

Accumulating evidence supports the ability of EphB6 to actively modulate cellular signaling pathways and be involved in the regulation of cellular responses. For example, several studies of EphB6 in human breast [37,38] and lung [39] cancer cells identify a direct effect of EphB6 on the activation of Erk kinases, whereas other studies suggest that EphB6 modulates Akt signaling in a more complex manner. For instance, interference with EphB6 activity was shown to promote Akt signaling in pediatric T cell acute lymphoblastic leukemia cells [40], but overexpression was found to enhance Akt activation in a mouse colonic adenoma cell line, IMCE ApcMin/+ [41]. Moreover, a recent study has suggested that the pro-survival effects of EphB6 on disseminated dormant estrogen receptor-positive breast cancer cells can be mediated by inhibition of the GSK3β pathway [42]. Examples of EphB6 effects on biological cellular responses include the inhibition of EphA2-mediated anoikis resistance in breast cancer cells by preventing Akt-mediated phosphorylation of the EphA2 receptor on Ser^897^ [43]. EphB6 also reduces motility and invasion of breast [36,37,44] and lung cancer cells [45,46] and partially reverses epithelial-to-mesenchymal transition traits of breast cancer cells [37]. In several malignancies, an inverse correlation between EphB6 expression and solid tumor aggressiveness was observed; these include melanoma [47], neuroblastoma [48,49], colorectal [50] and prostate [51] cancers, thereby suggesting that EphB6 is capable of suppressing invasive and metastatic phenotypes [52]. In a study by Truitt et al. (2010) [36], the propensity of EphB4 to drive the invasive behavior of breast cancer cell lines was attenuated by the expression of EphB6 such that the relative level of EphB4 and EphB6 determined its oncogenic potential. Consistent with these studies, EphB6 expression was shown to inhibit metastasis of lung [45] and colorectal [50] tumors in xenograft models. Interestingly though, despite its anti-invasive properties, EphB6 was also found to promote tumor initiation in breast cancer xenografts [37] and in a colorectal cancer model in the context of Apc mutations [41]. Together these findings suggest that given its ubiquitous tissue expression, promiscuous binding capabilities, lack of kinase activity and ability to attenuate the pro-metastatic capability of other Eph receptors, EphB6 is proposed to function as a switch that is capable of modulating the signaling output of Eph receptor clusters [36].

Phylogenetic analysis of EphB6 has identified that loss of kinase function is an evolutionarily “new” phenomenon by occurring solely in mammals [53]. This finding has given us a unique opportunity to compare the mammalian kinase-inactive EphB6 protein with the kinase-active EphB6 homolog from other non-mammalian species. Previous comparative analyses of EphB6 in the literature focused on the identity of EphB6 in comparison to other Eph RTKs of the same species. Although important in identifying the loss of kinase activity of the protein, more precise mechanistic aspects of EphB6 are more difficult to discern due to the profound differences in Eph members. For instance, human EphB6 is 49.7% identical to its closest human homolog EphB1 and 57.8% identical to the kinase-active EphB6 from the *Gallus gallus domesticus* (chicken) species. Likewise, the intracellular domain of EphB6 in humans is 51.8% identical with that of human EphB1, yet is 58.2% identical with the intracellular domain of chicken EphB6. Therefore, comparison of human EphB6 with the EphB6 of other mammals and non-mammals allows for an opportunity to more precisely identify residues that have co-evolved with the loss of kinase function to confer the new kinase-independent mechanisms of mammalian EphB6.

In this review, we postulate how mammalian EphB6 could function as a scaffold. To accomplish this, we will briefly describe what is currently known about the mechanistic action of kinase-active Eph receptors and how these functions are mediated at the amino acid/structural level and compare that to the amino acid sequence of both kinase-positive and kinase-negative EphB6 proteins from different species. With this analysis, we suggest that although mammalian EphB6 is kinase-negative, it has retained the allosteric regulatory mechanisms involving the juxtamembrane and the SAM domain linker that are used to regulate the kinase activity of kinase-active Eph receptors. Moreover, we suggest that the activation loop in EphB6 from placental mammals has evolved into an SH3 domain binding site whose conformational state is regulated by the activation state of the juxtamembrane domain. These analyses suggest that EphB6 has a switch-like mechanism of “active” and “inactive” states that regulate its ability to act as a scaffold that modulates functions of other Eph receptors in its cluster. How EphB6 modulates the activity of other receptors could involve an indirect mechanism by recruitment of adaptor proteins to the cluster that function to regulate the activity of kinase-active Eph receptors. Together, our analysis suggests that EphB6 is a modulator of Eph receptor cluster activity whose kinase-independent scaffold functions are precisely regulated.

## 2. Comparative Analysis of EphB6 Intracellular Domains

### 2.1. The (Pseudo) Tyrosine Kinase Domain

**Figure 2:**
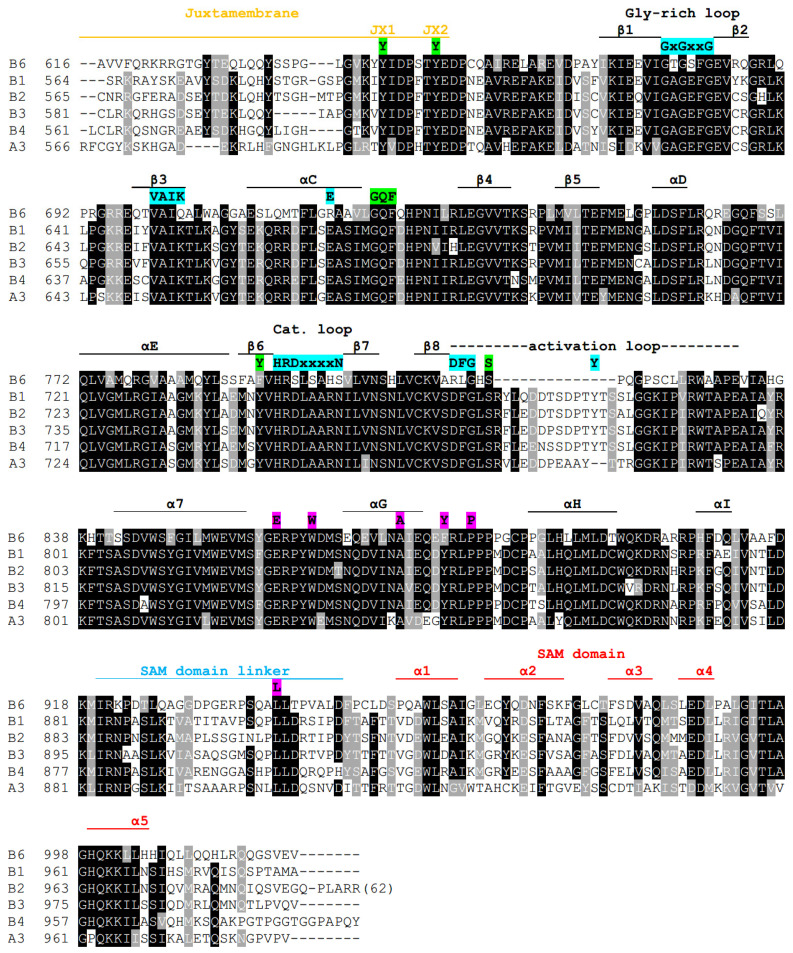
Comparative amino acid alignment of human EphBs and human EphA3 intracellular domains. Human EphB1 (P54762), EphB2 (P29323), EphB3 (P54753), EphB4 (P54760), EphB6 (015197) and EphA3 (P29320) are compared, and the location of domains are shown in different colored lettering above the alignment: juxtamembrane (orange), kinase (black), SAM domain linker (blue) and SAM domain (red). The identity and location of canonical amino acids that mediate Eph receptor mechanisms are shown in background coloring above the alignment: juxtamembrane allosteric regulation (green), kinase activity (violet), SAM domain linker allosteric regulation (pink). The secondary structure is based on the crystal structure of EphB2 (PDB:1JPA).

Tyrosine kinases utilize their kinase domain to catalyze the transfer of the γ phosphate of ATP to a tyrosine residue of a protein substrate. Kinases, in general, have a highly conserved architecture, being composed of two lobes designated the N-lobe and the C-lobe. The N-lobe is largely composed of β-sheet that, via several highly conserved regions, are used to bind to nucleotide and divalent cation cofactors. The C-lobe is primarily alpha-helical and houses both nucleotide and substrate binding sites. As shown in violet highlight coloring in Figure 2, the canonical ATP binding activity of kinases is accomplished by several domains: the glycine-rich loop that associates with nucleotide phosphate groups; the Lys of the VAIK domain in the β3 strand that interacts with ATP and is required for phosphoryl transfer; Glu of the αC-helix that maintains the lysine of the VAIK domain in the correct orientation for ATP-binding; and the DFG motif in the activation loop that also interacts with ATP. The substrate-binding functions of the kinase domain are governed in part by the Asp of the HRDxxxxN motif in the catalytic loop that aligns the substrate tyrosine for phosphorylation and Asn also of the catalytic loop that is required for catalysis. Kinases also house an activation loop that is used to occlude the kinase site from ATP and substrate until activation of the kinase domain.

**Figure 3:**
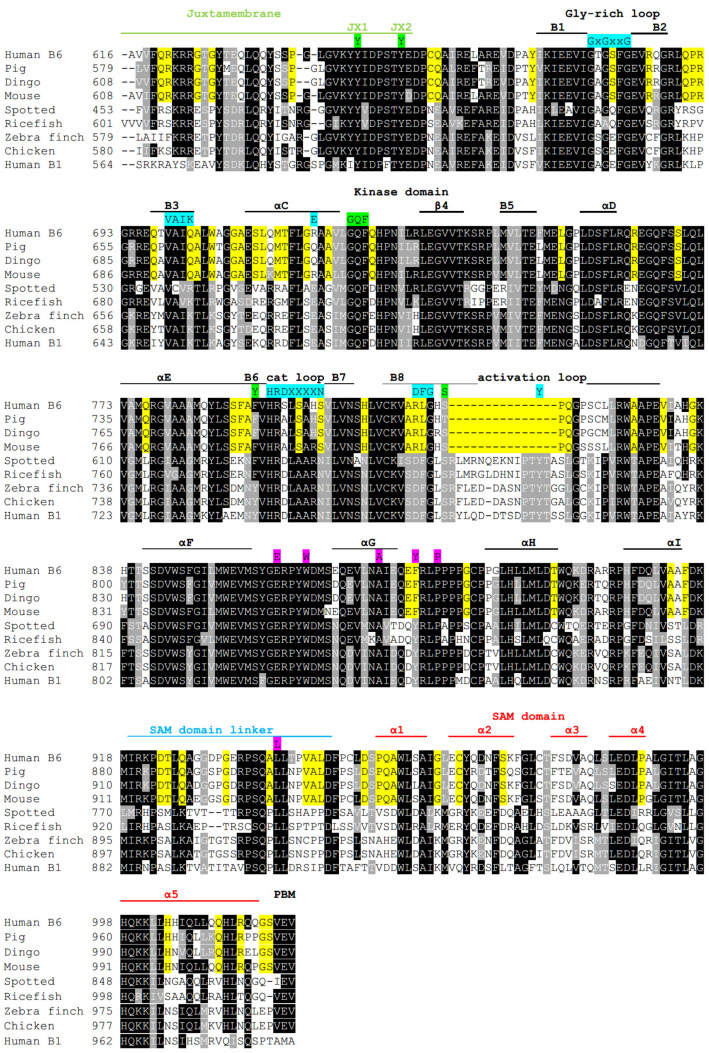
Comparative analysis of the EphB6 intracellular domain from placental mammalian species and non-mammalian species together with human EphB1. EphB6 from mammals: Human (O15197), Pig (F1SRT6), Dingo (XP_025289548.1) and Mouse (O08644) are compared to EphB6 from the fish species Spotted gar (W5MNT4) and Japanese ricefish (H2M8R4), and avian species Zebra finch (H0ZSI9) and Chicken (F1NAX7) together with human EphB1 (P54762). Refer to Figure 2 for the coloring of domains and amino acids that mediate mechanistic functions of Eph receptors. Residues that are conserved in mammalian EphB6 and are different than the equivalent position residue in non-mammalian EphB6 are shown in yellow background.

A comparison of human EphB6 with kinase-positive human EphB receptors (Figure 2) identifies that EphB6 contains the Glycine-rich loop but does not have the conserved Lys residue (K) of the VAIK domain nor the glutamate (E) of the αC helix. EphB6 also does not contain the Asp (D) and Asn (N) residues of the HRDxxxxN domain nor the Asp (D), Phe (F) and Tyr (Y) residues in the activation loop. A comparison of EphB6 between mammalian and non-mammalian species identifies the same dichotomy of amino acid conservation whereby non-mammalian EphB6 contain the Gly-rich loop, the Lys of the VAIK domain, the Glu of the αC helix and the HRDxxxxN, DFG and Tyr of the catalytic and activation loops and mammalian EphB6 members do not (Figure 3). This suggests that consistent with a previous phylogenetic study [53], EphB6 is kinase-negative in mammals only.

### 2.2. Mammalian EphB6 Retains Nucleotide-Binding Capacity

Although mammalian EphB6 houses several substitutions that have rendered it incapable of kinase activity, human EphB6 was shown to retain nucleotide-binding capability. A study by Murphy et al. (2014) [54] determined that significant deviations from the canonical ATP binding sites of kinases can still support nucleotide-binding capability, including in EphB6. In fact, mutation of the RLG (aa 813–815) motif in human EphB6 with the canonical DFG did not strengthen its nucleic acid binding capabilities but instead severely abrogated nucleic acid binding capability. In a study by Becher et al. (2013) [55], EphB6 could bind ATP-Mn and ATP-Mg at levels comparable to EphA3 and EphB2. This information has lead several recent reviews [56,57] to suggest that the nucleic acid binding activity of EphB6 could be used by the protein to induce conformational changes that are required to mediate specific interactions with other proteins. It is important to note that the conserved differences in EphB6 kinase domain sequences (Figure 3) between mammalian and non-mammalian species (K702Q, E719R/Q, N800S and D813R) are structurally mapped to the vicinity of the ATP binding pocket. This alteration of the ATP binding pocket may have a role in inducing a conformational change in EphB6.

Interestingly, retaining the ability to bind nucleotide to induce protein conformational changes that are required to interact with other proteins is a common strategy employed by many pseudokinases that act as molecular switches [18,19], including STRADα [58] and HER3 [59]. In a review by Hammaren et al. (2015) [60] the authors suggest that since many pseudokinases have not retained the phosphorylatable activation loop as a regulatory mechanism, the nucleotide-binding capability of the protein could be important for the regulation of conformations required for interaction with other proteins. Indeed, Shrestha et al. (2020) [57] postulate that the truncated activation loop amino acids of kinase-inactive EphB6 could form a protein interaction site. As shown in Figure 3, non-mammalian kinase-active EphB6 members retained the full length of the activation loop and its conserved Tyr residue, whereas the EphB6 activation loop was truncated in placental mammalian species and no longer contains the conserved Tyr (discussed further below). A homology modeling study of EphB6 in a study by Sheetz et al. (2020) [56] suggests that truncation of the activation loop rids the protein of the mechanism used by other Eph members to occlude the ATP-binding site until activation by phosphorylation. In fact, the authors suggest that RTK pseudokinases, in general, are either capable of nucleotide binding or have evolved to mimic an ATP-bound configuration.

### 2.3. Regulation of the Pseudokinase Domain

In addition to its ability to bind ATP, mammalian EphB6 has most likely retained the allosteric regulatory mechanisms involving the SAM domain linker and the juxtamembrane domain that are present in its kinase-active members.

#### 2.3.1. SAM-Linker Activation

C-terminal to the kinase domain is the SAM domain linker; this linker region functions to allosterically repress the C-lobe of the kinase domain via an interaction that involves a set of highly conserved residues (Figure 2 and Figure 3, background highlighted in pink) that tethers the SAM domain linker to the kinase domain. To accomplish this, a hydrophobic pocket created by residues Trp (W), Glu (E), Ala (A), Tyr (Y) and Pro (P) in the αF–αG loop, αG helix and αG–αH loop is used to retain a Leu residue in the SAM domain linker. Together these residues comprise the SAM linker network of residues required for allosteric regulation of the EphA3 kinase domain [61,62]. A comparison of human EphBs with each other identifies that all human EphBs contain these residues, including EphB6 (Figure 2), except that EphB6 has a Phe in place of the Tyr residue, a change that is also observed in EphA2 (not shown). Interestingly, kinase-negative EphB6 proteins have replaced this Tyr residue with Phe, whereas this residue has remained a Tyr in kinase-positive EphB6 proteins (Figure 3), possibly suggesting the importance of Tyr phosphorylation of this residue in kinase-positive Eph RTKs. Together, conservation of these residues suggests that both kinase-positive and kinase-negative EphB6 likely employ this mechanism of allosteric regulation.

The molecular mechanism used to disrupt allosteric repression by the SAM domain linker is not known; however, a study by Kwon et al. (2018) [62] suggests that since dimerization and oligomerization of Eph receptors are accomplished in part by the SAM domain, the interactions between SAM domains could disrupt the SAM domain linker from the C-lobe of the kinase domain, effectively releasing the negative allosteric regulation from the C-lobe. This interaction would constitute the initial step in the activation of the pseudokinase domain since the removal of the SAM domain linker would prime the juxtamembrane domain for autophosphorylation at the conserved tyrosine residues JX1 and JX2 as described below. In a study by Bulk et al. (2012) [46], deletion of amino acids 915–917 immediately upstream of the SAM domain linker of EphB6 (a deletion identified in non-small cell lung cancer patient samples) enhanced migration and metastatic capability of a non-small cell lung cancer cell line in a mouse model. Likewise, in a study by Yoon et al. (2019) [63], the authors found that mutations within the SAM domain linker of EphB6 including a Q926R mutation (Q926 is highly conserved in mammalian EphB6) or deletion of residues 915–917 confer resistance to paclitaxel in several cancer cell lines. They attributed this phenotype to the loss of SAM domain flexibility in EphB6 that abrogates the wild-type function of human EphB6, ultimately resulting in the lack of EphB6-mediated degradation of EphA2. Together, these studies identify the importance of the SAM domain linker in the function of human EphB6.

#### 2.3.2. The Juxtamembrane Domain

In kinase-active Ephs, the juxtamembrane region, when non-phosphorylated, functions to prevent both substrate and ATP from gaining access to the kinase domain [1]. It accomplishes this by locking the αC helix of the N-lobe in an inactive conformation that prevents phosphotransfer activity [64] and by sequestering the activation loop to the interior of the protein [61], a function that requires several conserved amino acids in the juxtamembrane and kinase domains (Figure 2 and Figure 3, highlighted in green). Derepression of the juxtamembrane domain is achieved by phosphorylation of two tyrosine residues designated JX1 and JX2 [64]. All human Ephs except EphA10 (not shown) contain the JX1 and JX2 tyrosine residues together with the “GQF” motif in the αC–β4 loop of the kinase domain. This GQF motif forms a pocket that houses the JX1 Tyr of the juxtamembrane domain when an Eph receptor is kept in its inactive autoinhibitory configuration, whereas the JX2 Tyr is solvent-exposed [61,62,64,65,66,67,68]. Derepression is initiated by sequential phosphorylation of the juxtamembrane domain involving the solvent-exposed JX2 Tyr initially, followed by the JX1 Tyr [62,67]. Following phosphorylation, both JX1 and JX2 can serve as binding sites for SH2 domain-containing proteins. These events activate the kinase domain and enable the Tyr in the activation loop to be accessible for phosphorylation, a modification that retains the kinase domain in its active state. Amino acid comparative analysis shows that both non-mammalian kinase-active EphB6 and the mammalian kinase-inactive EphB6 encode the JX1 and JX2 Tyr residues in the juxtamembrane domain together with the GQF motif of the αC–β4 loop (Figure 3, highlighted in green). As a result, conservation of canonical amino acids for juxtamembrane domain allosteric regulation of the N-lobe of the kinase domain in EphB6 suggests that mammalian kinase-negative EphB6 has also retained this mode of allosteric regulation.

### 2.4. Structural Organization of the Activation Loop

A study by Davis et al. (2008) [61] found that when EphA3 is in its inactive state, accomplished in part by interactions between the non-phosphorylated JX1 Tyr of the juxtamembrane domain and the GQF motif, steric hindrance between the JX1 Tyr and another Tyr residue at 742 exists (Figure 2 and Figure 3 highlighted in green). This hindrance prevents the activation loop from adopting an active conformation due to further steric hindrance between the hydroxyl group of Tyr^742^ with the hydroxyl group of Ser^768^ located in the activation loop (Figure 2 and Figure 3). Therefore the authors suggest that Tyr^742^ acts as an “activation sensor” for the activation loop, capable of transducing information about the state of the juxtamembrane domain to the activation loop irrespective of nucleotide-binding status. Interestingly when both the JX1 and JX2 Tyr residues were mutated to Phe, the kinase activity of EphA3 was abrogated, yet when Ser^768^ was also mutated to Ala, kinase activity was restored. Crystallographic analysis of the EphA3 triple mutant suggested that mutation of Ser^768^ to Ala alleviated the steric hindrance of Tyr^742^ and allowed the activation loop to take on a more ordered active conformation, hence allowing kinase activity to be re-established even when juxtamembrane repression remained. In mammalian EphB6, the Tyr^742^ equivalent residue in β6 was changed to Phe, whereas it was retained as a Tyr residue in kinase-positive EphB6 from avian species (Figure 3). However, in fish Eph receptors (that we suggest are kinase-positive), the Tyr residue was also switched to Phe, suggesting that Phe at this position could still maintain activation loop disorder in the absence of JX1 phosphorylation. This suggestion can be corroborated by the same Davis (2008) [61] study since in a JX1 Tyr to Phe and JX2 Tyr to Phe mutant EphA3 protein, additional mutation of Tyr^742^ to Ala allowed kinase activity to increase to nearly three-fold that of wild-type (activation loop disorder was reprieved), yet a Tyr^742^ to Phe mutation did not (kinase activity was approximately 1/4 of WT, activation loop remains disordered). This suggests that the Tyr to Phe substitution in β6 of human EphB6 likely supports the maintenance of activation loop disorder in the absence of JX1 tyrosine phosphorylation and that mammalian EphB6 has likely retained the steric hindrance effect on the activation loop when the juxtamembrane domain is unphosphorylated.

The next question, however, is why would EphB6 in mammals have retained this mechanism to regulate ordering of the activation loop? Since the activation loop is used to prevent ATP and substrate from gaining access to the kinase site when a kinase-positive Eph receptor is inactive, why would this mechanism be maintained in a kinase-negative version? We postulate that the “activation sensor” mechanism that links the juxtamembrane domain with the activation loop in kinase-positive Eph receptors is used by kinase-negative EphB6 to regulate access to the activation loop because it was repurposed into an SH3 domain binding site that is used to mediate interactions with adaptor proteins.

### 2.5. Alteration of the Activation Loop of Mammalian EphB6-SH3 Domain Binding Site

**Figure 4:**
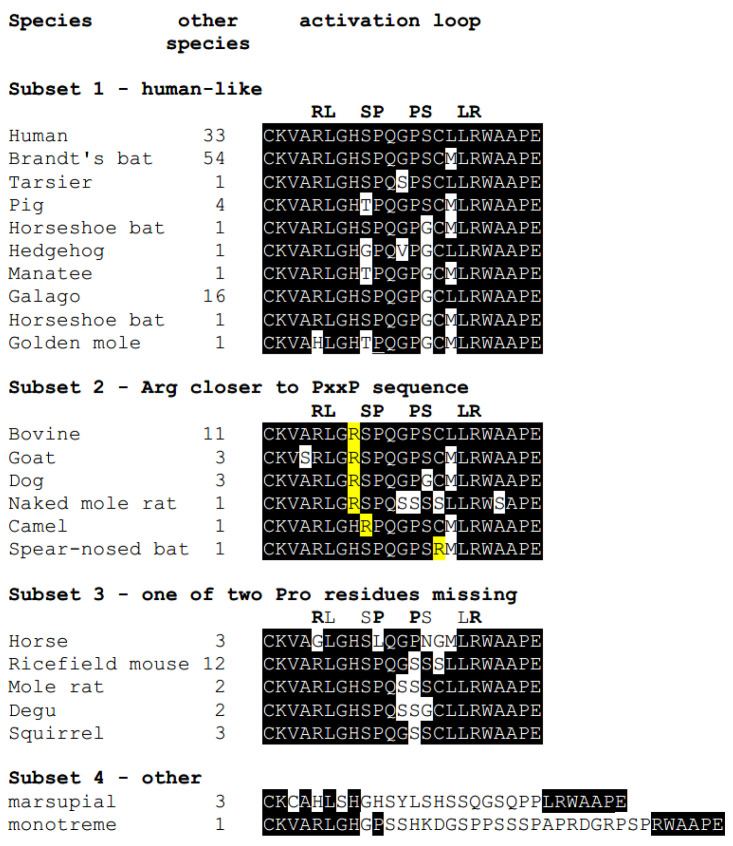
The activation loop of EphB6 from 155 different placental mammalian species, 3 marsupial mammalian species (opposum) and 1 monotreme mammalian species (Duckbill platypus). The two-fold symmetrical sequence that constitutes the putative SH3 domain binding site of human EphB6 is shown in bold and used for comparison with the activation loop from different mammalian species. “Other species” refers to the number of EphB6 proteins of other species that were sequenced to date that have an activation loop that is identical to the one given. Comparison of these sequences identifies 4 subsets; human-like, Arg closer to the PxxP sequence, one of two Pro residues missing and other.

Kinase-active Eph RTKs (and EphA10) have an approximate 35 amino acid activation loop that contains a conserved Tyr residue that, when phosphorylated, serves as an SH2 domain binding site to keep the activation loop in a configuration that prevents it from occluding access of ATP and substrate to the kinase site [69] (Figure 2 and Figure 3). Mammalian EphB6, however, has evolved a shorter activation loop (15 amino acid truncation) that does not include a Tyr residue. We suggest that the truncated mammalian EphB6 activation loop has been repurposed into an SH3 domain binding site since inspection of the human EphB6 activation loop identified a PxxP motif that is part of a two-fold symmetrical sequence RLxxSPxxPSxxLR that is conserved in the activation loop of most placental mammalian EphB6 members sequenced to date (Figure 3 and Figure 4). The SH3 domain is one of the most common domains in nature and is routinely used in conjunction with other domains (including SH2 domains) to mediate protein–protein interactions. The PxxP motif of the SH3 domain binding site commonly creates a left-handed polyproline (PPII) secondary structure that possesses two-fold rotational pseudo-symmetry, allowing SH3 domains to bind to it from both orientations. In addition to the PxxP binding site, canonical SH3 binding sites contain a positively charged Arg, Lys or His residue termed the orientation residue that is located on either side of the PxxP motif and is used to orient the SH3 domain to the PxxP motif [70,71,72,73]. Although two classes of PxxP motifs were initially characterized with class I motifs having the consensus sequence RxxPxxP(+) and class II with the sequence PxxPxR(−) [74], recent studies showed that SH3 binding sites are significantly diverse. In a study by Teyra et al. (2017) [75], the authors used peptide phage display to characterize the binding specificity of 115 SH3 domains and, in doing so, characterized seven additional classes, including the PxxxxR class of SH3 domain binding sites that would satisfy part of the RLxxSPxxPSxxLR putative SH3 binding motif. In addition, the authors found that approximately half of the SH3 domains exhibited non-canonical binding specificities (not class I or II) or multiple binding specificities.

The presence of Arg residues at the beginning and end of the RLxxSPxxPSxxLR sequence suggests that these residues could serve as orientation residues for the PxxP motif. However, Arg^813^ is the first amino acid in the symmetrical RLxxSPxxPSxxLR sequence and was previously shown in human EphB6 to be integral for nucleotide-binding since an R813D mutation abrogated nucleic acid binding capabilities [54]. This could suggest that Arg^813^ is not the orientation residue of the putative SH3 domain binding site, and instead, a histidine residue located nearer to the PxxP motif (His^816^) could serve this purpose (Figure 4). Although His is not as common as Arg or Lys in being the orientation residue for SH3 domains, His^816^ is highly conserved in mammalian EphB6 and in several species has been altered to an Arg residue, further supporting the potential importance of this position in SH3 domain binding recognition (Figure 4). Indeed there exists some heterogeneity in the putative SH3 domain binding site within the activation loop of mammalian EphB6 proteins since comparison of the activation loop from 155 EphB6 mammalian proteins shows some variability in this site. Most of the alterations are minor variations in the human sequence (subset 1), but there are several examples whereby the Arg residue has evolved to be closer to the PxxP sequence (subset 2). In addition, some activation domains evolved to rid themselves of one of the proline residues in the PxxP motif (subset 3), suggesting a preference for one binding orientation. Interestingly, in marsupial and monotreme mammalian species, the activation loop is significantly different from that of placental mammals. The marsupial mammalian activation EphB6 activation loop has retained a Tyr residue that is present in kinase-active EphB6 proteins from non-mammals, and the platypus (monotreme mammal) EphB6 activation loop is not truncated but is highly proline-rich. Together we suggest that the activation loop of placental mammalian EphB6 has evolved into an SH3 domain binding site. Experimental validation by identification of potential binding partners and determination of which amino acids serve to orient the SH3 domain requires investigation.

### 2.6. The EphB6 Switch Mechanism

In a manner similar to other pseudokinases, EphB6 is considered to act as a molecular switch that is capable of modulating the signals generated by an Eph receptor cluster. One of the methods EphB6 could use to perform this function is by recruiting proteins such as kinases, phosphatases, proteases or ubiquitinase ligases (or indirectly through interaction with their adaptor proteins) that modulate the phosphorylation state and thus kinase activity of individual members in the cluster. Retainment of allosteric regulation by the SAM domain linker and juxtamembrane allosteric regulation of an activation loop that has evolved to house an SH3 domain binding site suggests that the potential molecular switch function involving the SH3 domain binding site of mammalian EphB6 is a regulated process that occurs upon activation. We suggest that upon activation, phosphorylated Tyr residues in the juxtamembrane that serve as SH2 binding sites together with the SH3 domain binding site are used by mammalian EphB6 to recruit specific proteins that in turn modulate the signals generated by the Eph cluster. Repurposing the activation loop of a kinase domain into a protein interaction site is a strategy that was previously characterized for the pseudokinase STRADα ([58,76]. Upon activation (that requires the binding of ATP), its repurposed activation loop adopts an extended conformation that is used to mediate an interaction with LKB1 (reviewed by [77]).

By recruiting adaptor proteins to the Eph cluster, EphB6 could function by decreasing the phosphorylation state and thus kinase activity of individual kinase-active members in a cluster. A study by Shintani et al. (2006) [78] identified that protein tyrosine phosphatase receptor type O (Ptpro) is capable of dephosphorylating the JX1 and JX2 phosphate of both EphA- and EphB-type receptors. Likewise, a study by Akada et al. (2014) [43] proposed that EphB6 recruits a phosphatase that dephosphorylates residue Ser^897^ of EphA2. In addition, protein tyrosine phosphatase activity was implicated as the switch that regulates the cellular response to ephrin from repulsion to adhesion [79]. Employing such a molecular switch mechanism would be beneficial to modulate the activity of an Eph cluster without having to degrade it, thereby allowing the activity of previously assembled Eph clusters to be regulated in response to extracellular signals.

The molecular switch mechanism of EphB6 may also include promoting the degradation of Eph clusters by recruiting ubiquitin ligases since a functional relationship between EphB6 and the ubiquitin ligase c-Cbl was previously identified [33]. In the study by Yoon et al. (2019) [63] mentioned above, the effect of SAM domain linker (amino acids 901–929) mutations in conferring paclitaxel resistance in several cancer cell lines were attributed to the inability of mutated EphB6 to recruit the ubiquitin ligase c-Cbl to EphB6-EphA2 protein clusters. The inability to recruit c-Cbl by EphB6 meant that heightened levels of EphA2 remained active in the cell because they had evaded c-Cbl-mediated degradation. The authors suggest that mutation of residues 901–926 within EphB6 reduces the flexibility of the SAM domain such that c-Cbl cannot be recruited. The role of c-Cbl in EphB6-mediated signaling was also described in a study by Truitt et al. (2010) [36] whereby an interaction between c-Cbl and EphB6 (either directly or indirectly) required EphB6 phosphorylation that was provided by EphB4 upon stimulation with the ligand ephrin-B2. Although EphB6 could interact with c-Cbl directly, there are several c-Cbl adapter proteins that contain both SH2 and SH3 domains, including SLAP2 [80] and Grb2, that could also mediate EphB6–c-Cbl interactions.

## 3. Conclusions

**Figure 5:**
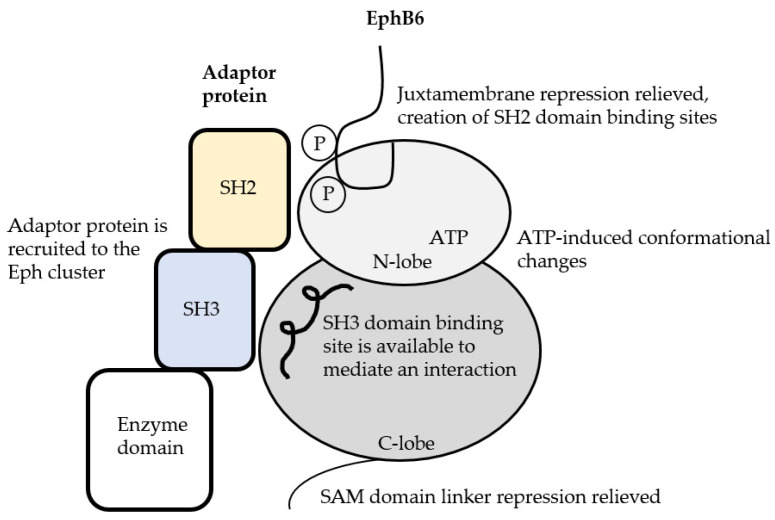
Hypothetical activation mechanism and function of mammalian EphB6. Activation could be initiated once allosteric regulation involving the SAM domain linker and the juxtamembrane domain are relieved. Phosphorylation of the juxtamembrane domain JX1 and JX2 Tyr residues creates SH2 domain binding sites and relieves repression of the activation loop. Since we suggest that the activation loop of mammalian EphB6 was repurposed into an SH3 domain binding site, this motif would now be in a configuration that allows it to work in conjunction with phosphorylated Tyr residues in the juxtamembrane domain to recruit adaptor proteins that contain SH3 and SH2 domains, respectively, to the Eph cluster. Given that mammalian EphB6 has retained an ability to bind nucleotide, it could be that ATP, when bound by EphB6, is used to induce conformational changes in the protein that may be required for its capability to recruit adaptor proteins, a possibility that nonetheless will require experimental investigation.

Our comparative analysis of mammalian kinase-negative EphB6 with its kinase-positive counterpart in non-mammals has provided insight into the potential mechanistic functions of the human pseudokinase EphB6. Instead of being an “inert” Eph receptor that solely prevents kinase-active Eph receptors from activating each other, we propose that EphB6 in mammals is a highly regulated pseudokinase whose function as a scaffold to recruit adaptor proteins to an Eph cluster is precisely controlled. To accomplish this, we suggest that mammalian EphB6 would require deactivation of the same allosteric regulatory mechanisms that are used to regulate the kinase activity of kinase-active Eph receptors involving the SAM domain linker and the juxtamembrane domain (Figure 5). Derepression of the juxtamembrane domain of EphB6 by phosphorylation could release the steric hindrance imposed at the activation loop, allowing the repurposed SH3 binding domain to adopt a configuration that together with SH2 domain binding sites (composed of phosphorylated Tyr residues in the juxtamembrane domain) are used to recruit adaptor proteins to EphB6. Given that EphB6 has retained the ability to bind nucleotide, an added level of control could exist with nucleotide binding, such that the binding of ATP is required to induce conformational changes that affect its interaction with other proteins, including adaptor proteins. Therefore in this fashion, mammalian EphB6 would employ a complex mechanism to activate its function as a scaffold, ensuring that its function to modulate the signals generated by an Eph receptor cluster is precisely regulated and used when required.
